# Comparison of Regulations for Arsenic and Heavy Metals in Herbal Medicines Using Pharmacopoeias of Nine Counties/Regions

**DOI:** 10.1007/s43441-023-00532-2

**Published:** 2023-05-18

**Authors:** Isa Inada, Fumiyuki Kiuchi, Hisashi Urushihara

**Affiliations:** 1 Division of Drug Development and Regulatory Science, Faculty of Pharmacy, Keio University, 1-5-30 Shibakoen, Minato-Ku, Tokyo, 105-8512 Japan; 2 Division of Natural Medicines, Faculty of Pharmacy, Keio University, 1-5-30 Shibakoen, Minato-Ku, Tokyo, 105-8512 Japan

**Keywords:** Heavy metal, Herbal medicine, Pharmacopoeia, Quality standard, Elemental impurity, Harmonization

## Abstract

**Introduction:**

Standardization is an import factor in ensuring the safety, efficacy, and quality of herbal medicines, and facilitates their international commerce. Heavy metal poisoning due to herbal medicines has been reported in many countries. Here, to better understand the current state of harmonization, we compared regulations for arsenic and heavy metals in herbal medicines across seven countries and two regions with two international standards.

**Methods:**

We studied the monographs of herbal medicines of seven countries and two regions, as well as the WHO guidelines and ISO standards. We then compared the limits and test methods adopted for elemental impurities in herbal medicines listed in the monographs and standards among countries.

**Results:**

The number of herbal medicines assessed amounted to over 2000. Limits and test methods adopted for elemental impurities in herbal medicines varied by country/region and organization. Although WHO recommends a uniform upper limit for lead and cadmium for all herbal medicines, some countries set unique upper limits for individual herbal medicines. ISO 18664:2015 lists only instrumental analysis methods, whereas Japan and India list only chemical methods.

**Conclusions:**

Many countries do not adhere to the WHO or ISO recommendations on elemental impurities in herbal medicines. These findings suggest the presence of differences in regulations for herbal medicines among countries/regions, likely rooted in cultural differences and policies aimed at maintaining the diversity of herbal medicines. Regulatory convergence by “loose harmonization” to internationally agreed standards appears a feasible option to maintain diversity and safety, and promote international trade in herbal medicines.

**Supplementary Information:**

The online version contains supplementary material available at 10.1007/s43441-023-00532-2.

## Introduction

The World Health Organization (WHO) has defined traditional medicine as a medical science with a long history and “the sum total of the knowledge, skill, and practices based on the theories, beliefs, and experiences indigenous to different cultures” [1]. Following recent international attention, Traditional Chinese Medicine (TCM) was added to the 11th revision of the International Classification of Diseases (ICD-11), which was approved in 2019 [2].

To ensure the safety, efficacy, and quality of traditional medicines which have come to be widely used internationally, expansion of their knowledge base and strengthening of relevant regulations are essential [3]. Accordingly, WHO launched the International Herbal Pharmacopoeia project in 2020 [4].

In the field of foods and medicines, elemental impurity is a significant concern for human health. International harmonization of standards for elemental impurities is being pursued through the Codex Alimentarius Standard [5] and the International Council for Harmonization of Technical Requirements for Pharmaceuticals for Human Use (ICH) initiative. The Codex standards were developed as an international standard for foods by the Codex Alimentarius Commission, established in 1963. The Agreement on the Application of Sanitary and Phytosanitary (SPS) Measures [6] requires that member countries/regions base their SPS measures on the Codex standards for international trade. It also recommends that member countries/regions harmonize domestic standards for foods with the Codex standards. Regarding pharmaceuticals, local regulations for elemental impurities are harmonized by ICH Harmonised Guideline: Guideline for elemental impurities (ICH-Q3D), except for herbal medicines, biological products, and the like [7]. In 2018, the United States Pharmacopeial Convention replaced the heavy metals limit test in the US pharmacopoeia, which quantifies the total amount of heavy metals by colorimetry, with the elemental impurities test, which quantifies individual heavy metals [8]. Similarly, European Pharmacopoeia Commission decided not to recommend the heavy metal limit test for drugs used in humans [9]. The Japanese health authority is also planning to implement regulations based on ICH-Q3D in the 18th revision of the Japanese Pharmacopoeia [10].

With regard to herbal medicines, several cases of heavy metal poisoning have been reported in many countries from the 1990s [11, 12]. A case of lead poisoning caused by the hypoglycemic agent “Zhen Qi Jiang Tang” was reported in Japan. Two men were hospitalized and diagnosed with lead poisoning among about 150 patients with type 2 diabetes taking the unapproved Zhen Qi Jiang Tang product in 1998 [12]. China proposed “Heavy Metals in Natural Materials used in Traditional Chinese Medicine” to TC249, an expert committee on TCM established by the International Organization for Standardization (ISO) in 2009. The proposal called for individual assay of each metal and determination of uniform upper limits for all herbal medicines [13]. In terms of safety, however, the question of whether provisions on upper limits should be left to the discretion of each country or be harmonized has aroused heated debate [14].

Herbal medicines have been used regionally and developed independently based on clinical experiences in each region. Therefore, standardization is highly challenging, as discussed in the fourth Conference for Trilateral Communication between East Asian Pharmacopoeia Committees on Natural Medicines (TEAPN), held in 2019 to share information on pharmacopoeias from Japan, China, and Korea [15]. Further, Japan and Korea expressed concern over ISO’s establishment of TC249, namely that standardization would result in the loss of diversity of herbal medicines, leading in turn to a decrease in treatment options [13]. WHO recommends that each country should set regional or national standards for the maximum amounts of heavy metals in their guidelines, while also stating that international harmonization of heavy metal regulations would be desirable [16]. International harmonization of regional/national regulation is critical for facilitating international trade. On the other hand, the cultural and medical value of preserving diversity in herbal medicines in local medical practice and society should be taken into consideration.

Despite safety concerns for herbal medicines, literature on regulatory provisions for quality control of herbal medicines for international comparison is scarce. In this study, to better understand the current state of international harmonization, we reviewed regulations and guidelines concerning the amounts of elemental impurities in herbal medicines around the world.

## Material and Methods

### Definition and Scope

The elemental impurities investigated in this study included arsenic, lead, mercury, and cadmium, which are classified as class 1 elements in ICH-Q3D [17]. They are human toxicants that have limited or no use in the manufacture of pharmaceuticals. Trivalent or pentavalent arsenic, divalent mercury, and methylmercury were excluded.

We adopted the definition of herbal medicines provided in the Japanese Pharmacopoeia. The “General rules for crude drugs” of the Japanese Pharmacopoeia defines herbal medicines (crude drugs) as medicinal parts obtained from plants or animals, cell inclusions, and minerals. In other words, we defined them as raw material and excluded any preparations and compounded medicines. Although powdered herbal medicines to which the same regulations apply were excluded, processed herbal medicines were considered as separate items and included. The amount of elemental impurities contained in herbal medicines may change due to processing, and this may be reflected in differences in the regulations. We included all mineral-derived herbal medicines (mineral medicines) because these contain significantly large amounts of elemental impurities [18] and are considered important in comparing standards for elemental impurities.

### Study Materials

**Figure 1:**
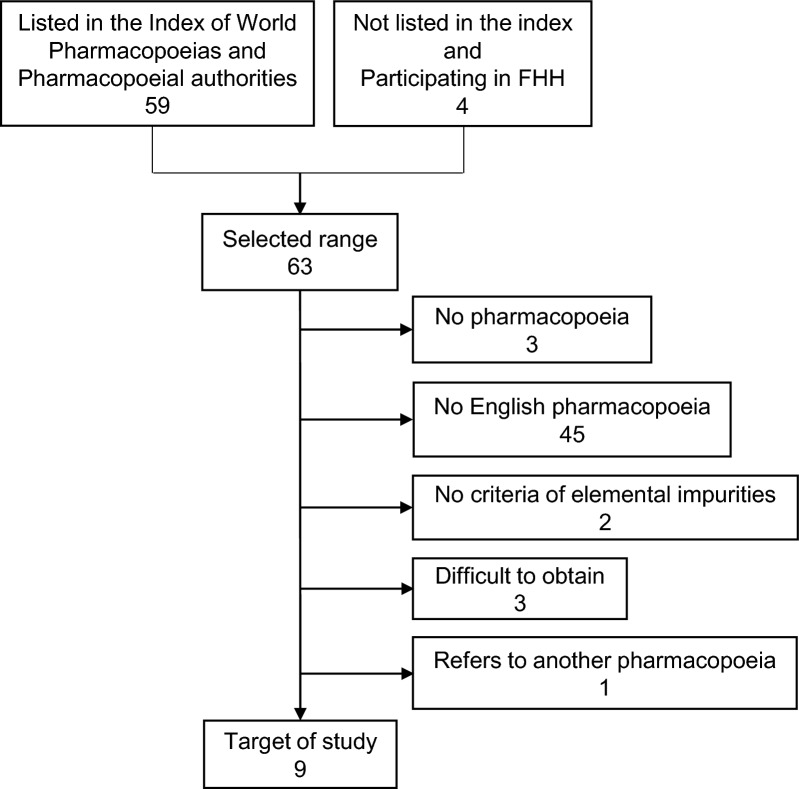
Flowchart of selection of target countries and regions for study. *FHH* Forum for the Harmonization of Herbal Medicines.

We searched the documents from WHO, ISO, Brazil, China, Europe, Hong Kong, India, Japan, Korea, United States, and Vietnam. These countries and regions were selected from among participants in the Forum for the Harmonization of Herbal Medicines (FHH) (Table S1) or from the list in the Index of World Pharmacopoeias and Pharmacopoeial Authorities [19] (Fig. 1). We excluded some countries and regions which met one or more of the exclusion criteria shown in Table S2.

Table 1 shows the latest versions of the documents as of December 2021 and abbreviations used for the study.

**Table 1:** Document names and abbreviations

Country/region	Document name	Abbreviation
Brazil	Brazilian Pharmacopoeia 6th edition	BP6
China	Pharmacopoeia of the People’s Republic of China 2020	CP2020
Europe	European Pharmacopoeia 10th edition (10.7)	EP10
Hong Kong	Hong Kong Chinese Materia Medica Standards Vol.1 ~ 10	HKCMMS
India	Indian Pharmacopoeia 2018	IP2018
Japan	Japanese Pharmacopoeia 18th edition	JP18
Korea	Korean Pharmacopoeia 12th edition	KP12
United States	United States Pharmacopeia and National Formulary 2020	USP43-NF38
Vietnam	Vietnamese Pharmacopoeia 5th edition	VP5

International Organization	Document name	
ISO	ISO 18664:2015 “Traditional Chinese Medicine – Determination of heavy metals in herbal medicines used in Traditional Chinese Medicine”	
WHO	WHO Monographs on Selected Medicinal Plants Vol.1 ~ 4 WHO guidelines for assessing quality of herbal medicines with reference to contaminants and residues	

We selected pharmacopoeias listed in the Index of World Pharmacopoeias and Pharmacopoeial Authorities [19]. In cases where multiple pharmacopoeias were listed, we selected the major pharmacopoeia that listed herbal medicines. Hong Kong, which was not listed in the Index of World Pharmacopoeias and Pharmacopoeial Authorities, did not have a pharmacopoeia, and we used corresponding documents instead. For international guidelines, the WHO guidelines on herbal medicines and the ISO standards were searched for stipulations on elemental impurities in herbal medicines. Only the ISO/TC249 standards for test methods were used, because many of the standards for individual herbal medicines were still under development and specific upper limits for elemental impurities had not been set.

### Analysis Methods

The herbal medicines listed in the study documents in Table 1 were investigated except for ISO 18664:2015, which contains a guideline for test methods only. The number of monographs with elemental impurity stipulations and the test methods were summarized for each study document.

We compared the limits of elemental impurities and the total amount of heavy metals in the monograph of documents in Table 1, which are summarized by bubble plot. Figures were created by JMP Pro 16 (SAS Institute Inc, NC, USA).

## Results

### Elemental Impurity Stipulations

The chapters of monographs listing herbal medicines in the covered documents are shown in Table 2.

**Table 2:** Title of monograph chapters on herbal medicines

Document name	Title of monograph chapters
BP6	Plants Medicines
CP2020	Chinese Materia Medica and prepared slices of Chinese crude drugs
EP10	Herbal drugs and herbal drug preparations Homeopathic Preparations
IP2018	Herbs and Herbal Products
JP18	Crude drugs and related drugs
KP12	Herbal drugs and herbal drug preparations
USP43-NF38	USP Monograph NF Monograph Dietary Supplement
VP5	Materia Medica

The number of listed herbal medicines in each document ranged from 83 in Brazilian Pharmacopoeia 6th edition (BP6) to 611 in Pharmacopoeia of the People’s Republic of China 2020 (CP2020), and most were animal- or plant-origin herbal medicines (animal/botanical medicines). BP6, European Pharmacopoeia 10th edition (EP10), Indian Pharmacopoeia 2018 (IP2018), and WHO Monographs on Selected Medicinal Plants did not contain mineral medicines (Table 3).

**Table 3:** Number of listed herbal medicines in each document

Document name	Number of listed herbal medicines	Number of animal/botanical medicines	Number of mineral medicines
BP6	83	83	–
CP2020	611	587	24
EP10	254	254	–
HKCMMS	330	322	8
IP2018	90	90	–
JP18	173	167	6
KP12	165	164	1
USP43-NF38	103	99	4
VP5	330	328	2
WHO	117	117	–

*WHO* WHO Monographs on Selected Medicinal Plants

**Figure 2:**
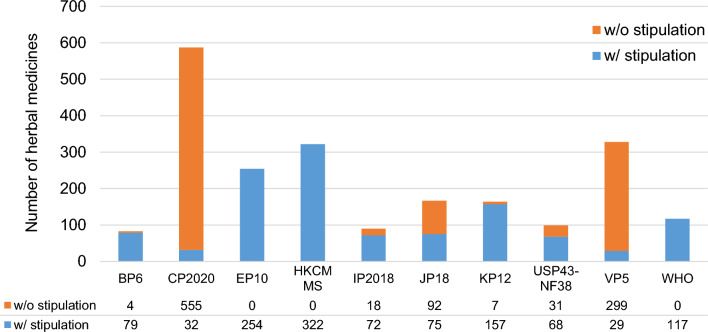
Number of animal/botanical medicines with and without stipulations. *WHO* WHO Monographs on Selected Medicinal Plants; *w/o* without; *w/* with.

As shown in Fig. 2, the number of animal/botanical medicines listed ranged from 83 items in BP6 to 587 items in CP2020. EP10 (254 items), Hong Kong Chinese Materia Medica Standards (HKCMMS, 322 items), and WHO Monographs on Selected Medicinal Plants (117 items) stipulated impurities for all animal/botanical medicines. As EP10 and the WHO Monographs on Selected Medicinal Plants contained only monographs of animal/botanical medicines, all listed herbal medicines in these documents had these stipulations. For elemental impurities, in contrast, about 95% (555/587) of animal/botanical medicines in CP2020 and about 91% (299/328) in Vietnamese Pharmacopoeia 5th edition (VP5) did not have stipulations. BP6, IP2018, Korean Pharmacopoeia 12th edition (KP12) and United States Pharmacopoeia and National Formulary 2020 (USP43-NF38) provided stipulations of impurities for most of the animal/botanical medicines (95%, 80%, 95%, and 69%, respectively). Japanese Pharmacopoeia 18th edition (JP18) provided stipulations for less than half of the listed herbal medicines (44.9%).

**Figure 3:**
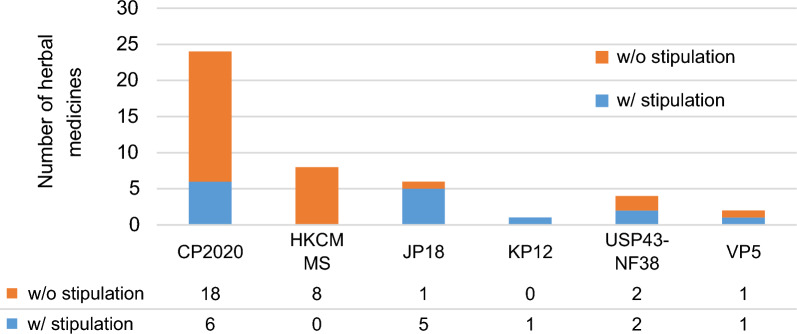
Number of mineral medicines with and without stipulations. *w/o* without; *w/* with.

Figure 3 shows the numbers of mineral medicines with and without stipulations for elemental impurities. The number of mineral medicines listed ranged from 1 item in KP12 to 24 items in CP2020. In contrast to the animal/botanical medicines, no mineral medicines had stipulations for elemental impurities in HKCMMS. About 75% of mineral medicines in CP2020 did not have stipulations. JP18 provided stipulations for impurities for most of mineral medicines (83%). USP43-NF38, VP5, and KP12 provided small numbers of stipulations for mineral medicines. No other study documents had stipulations for impurities in mineral medicines.

### Upper Limits of Elemental Impurities

WHO, BP6, and HKCMMS have set uniform upper limits for all listed herbal medicines. EP10 and KP12 have set uniform upper limits, with exceptions for some herbal medicines. CP2020 and VP5 have set varying upper limits for each element and total heavy metals only for small fractions of listed herbal medicines. EP10, HKCMMS, and USP43-NF38 specified the individual determination of heavy metals only, whereas IP2018 and JP18 specified upper limits for arsenic and total heavy metals only, and did not specify the individual determination of elements other than arsenic. For mineral medicines, most documents did not specify the individual determination of heavy metals.

**Figure 4:**
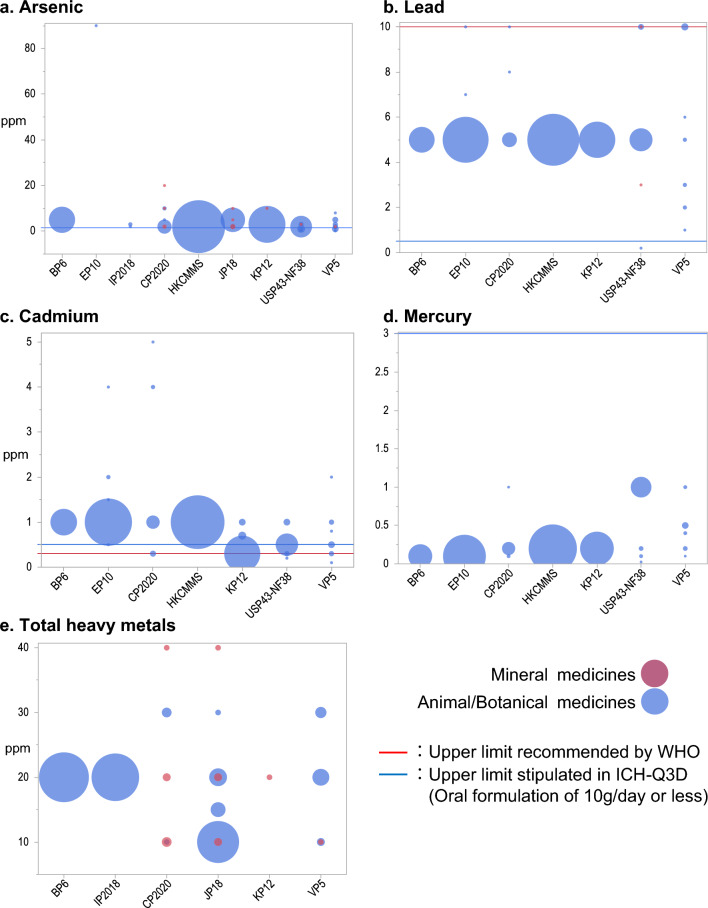
Comparison of the upper limits of elemental impurity. BP6, EP10, and IP2018, which did not list mineral medicines, are at left; the other documents that listed both animal/botanical medicines and mineral medicines are at right. The Y-axis indicates the upper limits of the corresponding elemental impurities. The center position of a bubble indicates the upper limit value and its size indicates the number of herbal medicines whose upper limit was set at that value. The reference lines represent the limits recommended in the WHO guidelines and the upper limits stipulated in ICH-Q3D, which define the limits of common concentrations of elemental impurities contained in the daily oral preparations of 10 g or less.

- The upper limit of arsenic
  - ICH recommended 1.5 ppm for arsenic in ICH-Q3D, and the ranges of the upper limits stipulated for many animal/botanical medicines in the study documents were close to this value.
  - For animal/botanical medicines, BP6 has set 5 ppm for all 83 items, HKCMMS has set 2 ppm for all 322 items, and KP12 has set 3 ppm for all 157 items (Fig. 4a).
  - CP2020, JP18, USP43-NF38, and VP5 have set varying upper limits ranging from 0.2 to 20 ppm.
  - EP10 stipulated an upper limit of arsenic only for Kelp (90 ppm).
  - For mineral medicines, CP2020, JP18, KP12, USP43-NF38, and VP5 had stipulations of upper limits for arsenic.
  - The upper limits for mineral medicines were rarely uniform, and included higher values than those for most animal/botanical medicines.
- The upper limit of lead
  - The upper limits of lead mostly ranged from 0.5 ppm stipulated in ICH-Q3D to 10 ppm recommended by WHO, and no items exceeded the WHO recommendation (Fig. 4b).
  - An upper limit of 5 ppm was set for all 83 items in BP6, all 322 items in HKCMMS, and all 157 items in KP12 for animal/botanical medicines.
  - Also, EP10, CP2020, and USP43-NF38 have set an upper limit of 5 ppm for most animal/botanical medicines (253/254, 26/28, and 63/68, respectively).
  - VP5 has set varying upper limits ranging 1 to 10 ppm.
  - USP43-NF38 has set upper limits for lead in mineral medicines, namely 10 ppm for Kaolin and 3 ppm for Ground Limestone. No other study documents stipulated upper limits for mineral medicines.
- The upper limit of cadmium
  - Upper limits of cadmium for most herbal medicines were above the limits recommended by WHO (0.3 ppm) and stipulated in ICH-Q3D (0.5 ppm, Fig. 4c).
  - BP6 and HKCMMS have set 1 ppm as the upper limit for all 83 and 322 items of animal/botanical medicines, respectively.
  - EP10 has set an upper limit of 1 ppm for most items (251/254).
  - CP2020 and VP5 have set varying upper limits, with the highest limits of 5 and 2 ppm, respectively.
  - KP12 and USP43-NF38 have set 0.3 ppm and 0.5 ppm for most items, respectively (145/157 and 56/65, respectively).
  - No study documents stipulated upper limits of cadmium for mineral medicines.
- The upper limit of mercury
  - All upper limits were below the upper limit of mercury stipulated in ICH-Q3D (3 ppm, Fig. 4d).
  - For animal/botanical medicines, BP6 has set 0.1 ppm for all 83 items, EP10 has set 0.2 ppm for all 254 items, HKCMMS has set 0.2 ppm for all 322 items, and KP12 has set 0.2 ppm for all 157 items.
  - CP2020, USP43-NF38, and VP5 have set varying upper limits, ranging from 0.2 to 1 ppm. CP2020 and USP43-NF38 have set upper limits of 0.2 ppm and 1 ppm for most items, respectively (24/27 and 59/65, respectively).
  - No study documents stipulated upper limits for mercury in mineral medicines.
- The upper limit of total heavy metals
  - WHO and ICH-Q3D did not provide recommendations for upper limits of total heavy metals.
  - The upper limits for mineral medicines varied more widely than those for animal/botanical medicines (Fig 4e).
  - A unique upper limit of 20 ppm for total heavy metals were set in BP6 and IP2018 for all listed herbal medicines
  - JP18 stipulated varying upper limits for all listed herbal medicines, ranging from 10 to 40 ppm.
  - CP2020 stipulated varying upper limits for a fraction of listed herbal medicines, ranging from 10 to 40 ppm.
  - KP12 has set 20 ppm for Longgu, the only mineral medicine for which it set an upper limit for elemental impurities.
  - CP2020, KP12, and VP5 specified similar upper limits for the same items, particularly mineral medicines.

### Types of the Test Method Used to Evaluate Elemental Impurities

We tabulated the test methods used in the documents in Table 4. All of the test methods listed in ISO 18664:2015 utilized analytical instrumentation, including Atomic Absorption Spectrometry (AAS), Inductively Coupled Plasma Atomic Emission Spectrometry (ICP-AES), Inductively Coupled Plasma Optical Emission Spectrometry (ICP-OES), and Inductively Coupled Plasma Mass Spectrometry (ICP-MS). Chemical methods were not listed in ISO 18664:2015 but were adopted by JP18, KP12, CP2020, VP5, and IP2018 for arsenic, and by USP43-NF38 for arsenic and lead. Of these, only JP18 and IP2018 adopted only chemical methods for all herbal medicines. The chemical methods included the arsenic test, the lead test, and the heavy metal limit test. The heavy metal limit test involves the addition of sodium sulfide under weakly acidic conditions to colorize heavy metals and comparison of the color with a standard solution.

**Table 4:** Comparison of test methods used for evaluating elemental impurities

Element	Document name	Methods in ISO 18664:2015			Chemical method	Mercury analyzer
		AAS	ICP-AES ICP-OES	ICP-MS		
Arsenic	BP6	✓	–	–	–	–
	EP10	✓	✓	✓	–	–
	IP2018	–	–	–	✓	–
	CP2020	✓	–	✓	✓	–
	HKCMMS	–	–	✓	–	–
	JP18	–	–	–	✓	–
	KP12	✓	–	✓	✓	–
	USP43-NF38	–	✓	✓	✓	–
	VP5	✓	–	–	✓	–
Lead	BP6	✓	–	–	–	–
	EP10	✓	✓	✓	–	–
	CP2020	✓	–	✓	–	–
	HKCMMS	–	–	✓	–	–
	KP12	✓	–	✓	–	–
	USP43-NF38	–	✓	✓	✓	–
	VP5	✓	–	–	–	–
Cadmium	BP6	✓	–	–	–	–
	EP10	✓	✓	✓	–	–
	CP2020	✓	–	✓	–	–
	HKCMMS	–	–	✓	–	–
	KP12	✓	–	✓	–	–
	USP43-NF38	–	✓	✓	–	–
	VP5	✓	–	–	–	–
Mercury	BP6	✓	–	–	–	–
	EP10	✓	✓	✓	–	–
	CP2020	✓	–	✓	–	–
	HKCMMS	–	–	✓	–	–
	KP12	–	–	–	–	✓
	USP43-NF38	–	✓	✓	–	–
	VP5	✓	–	–	–	–

*AAS* Atomic Absorption Spectrometry; *ICP*-*AES* Inductively Coupled Plasma Atomic Emission Spectrometry; *ICP*-*OES* Inductively Coupled Plasma Optical Emission Spectroscopy; *ICP*-*MS* Inductively Coupled Plasma Mass Spectrometry

JP18, KP12, CP2020, VP5, IP2018, and BP6 provided a test method for total heavy metals and adopted the heavy metal limit test, although ISO 18664:2015 mentioned no test method for total heavy metal. The chapter “4.1 Heavy metals” of JP18 stipulated that AAS, ICP-AES/ICP-OES, and ICP-MS were applicable when the heavy metal limit test was not applicable due to turbidity or other reasons.

KP12 described use of a “Mercury analyzer” for the test of mercury but no specific analysis method was provided.

## Discussion

We compared regulations on arsenic and heavy metals in herbal medicines across seven countries and two regions with reference to the guidelines/monographs of two international organizations to overview the related regulations of each country and region and the current status of international harmonization. We found that international harmonization of national regulations on elemental impurities in herbal medicines in accordance with the guidelines/monographs of WHO/ISO documents has yet to be achieved.

The WHO guidelines recommended uniform upper limits for lead and cadmium, respectively, for all herbal medicines. However, CP2020, JP18, and VP5 made provisions that were tailored to each herbal medicine, and therefore the number of herbal medicines with stipulations was small. EP10 and HKCMMS adopted a similar uniform upper limit approach to WHO.

ISO 18664:2015 listed only instrumental analysis methods, which are expensive but allow individual determination of each element. Nevertheless, IP2018 and JP18 regulated no individual heavy metals, but their total amount that can be measured inexpensively by chemical methods.

### Stipulation for Animal/Botanical Medicines

CP2020 and VP5 had stipulations for elemental impurities for only a small number of animal/botanical medicines (Fig. 2). This may be because the collection and assay of herbal medicine samples to determine elemental impurities is time-consuming as China conducts assays using real samples of herbal medicines available in the market and uses actual measurements as a reference for determining their regulations [20]. VP5 has also set the various upper limits for individual items, suggesting that assays had been conducted for each herbal medicine.

In contrast, EP10 and HKCMMS had stipulations for elemental impurities for all herbal medicines with less variability in upper limits. This may have been due to the policy of EP10 and HKCMMS in setting a uniform upper limit for all herbal medicines without investigation for any individual herbal medicine. Similarly, the WHO guidelines also adopted a uniform upper limit for lead and cadmium, respectively, as shown in Fig. 4. EP10 has set a considerably high upper limit of 90 ppm arsenic for Kelp. It is reported that most of the arsenic contained in kelp is organic arsenic, including arsenobetaine and arsenosugar, which are generally considered to be less toxic than inorganic arsenic [21]. Therefore, a relatively high upper limit was set for the total amount of arsenic.

Upper limits set in HKCMMS were consistent with the upper limits specified in other documents for many herbal medicines, because the upper limits were set with reference to the pharmacopoeias of other countries and regions such as CP and EP [22].

### Stipulations for Mineral Medicines

Most of the documents specified the amount of total heavy metals for mineral medicines. Thus, arsenic needs to be assayed individually in addition to total heavy metal in mineral medicines, because arsenic is not heavy metal. It is not cost-effective to purchase expensive analytical equipment to assay arsenic only, and chemical methods were the choice for arsenic assay in most of the documents (Table 4).

HKCMMS did not set any stipulations on elemental impurities for all mineral medicines, in contrast to animal/botanical medicines (Figs. 2, 3). Compared to plants and animals, minerals naturally contain larger amounts of metal element [18]. HKCMMS might have been reluctant to set upper limits for elements contained in mineral medicines, due to concern that doing so might decrease the availability of mineral medicines.

USP43-NF38 specified the amount of lead for Kaolin and Ground Limestone but not the total amount of heavy metals. This is because the test method adopted in USP43-NF38 was individual assay for each element of herbal medicines. In addition, the United States Pharmacopeial Convention replaced the heavy metal limit test for measuring the total amount of heavy metals in the USP with the method of the ICH-Q3D (R1) guideline, which measures each heavy metal individually [8].

### Recommendation by International Organizations

The limit recommended by WHO, Acceptable Daily Intake (ADI), and that stipulated in ICH-Q3D, Permissible Daily Exposure (PDE), were used as reference values for upper limits [16, 17]. Although it was excluded from this study, upper limits for finished products in Thai Herbal Pharmacopoeia (THP) 2021 were set by referencing the ADI, and were therefore consistent with the recommendations of WHO [23]. ICH-Q3D provides regulations regarding elemental impurities that should not cause contamination during synthesis and processing in chemically synthesized drugs. However, the regulation is not applicable to herbal medicines and biologics. Herbal medicines are in principle administered after decoction, such as in the form of an extract, and thus differ from pharmaceuticals, which are ready for administration. Given that the residue of the decoction is discarded and only the extract is ingested, the upper limits of elemental impurity in raw herbal medicines could be somewhat higher than the thresholds required in PDE and ADI.

With respect to test methods, ISO may have regarded the individual determination of elemental impurities as of greater importance than the determination of the total amount of heavy metals, as indicated by the fact that ISO 18664:2015 did not describe a method for the determination of the latter.

### International Harmonization of Regulations on Elemental Impurities in Herbal Medicines

We found that two important aspects of the related regulations mentioned below differed among the countries and regions, and international standards.

1. Upper limits of elemental impurities are set uniformly for all herbal medicines or individually for each herbal medicine.
2. Upper limits of elemental impurities are set for individual elements or for total amount of heavy metals.

WHO and ISO have supported harmonization for elemental impurities by setting a uniform upper limit for all herbal medicines and stipulations for uniform thresholds for each element in their guidelines. Harmonization consistent with this process is considered important for facilitating and promoting international trade. Indeed, the two aspects above were major topics in the proposal “Heavy Metals in Natural Materials used in Traditional Chinese Medicine,” which was discussed in ISO/TC249/WG1 [13, 14].

1. Uniform Stipulations for all Herbal Medicines

Although WHO has set a uniform upper limit for all herbal medicines, it is in fact difficult to maintain the diversity of herbal medicines under uniform stipulations. Once a “one-size-fits-all” regulation is established, herbal medicines with deviation will be removed from the market without considering variability in the actual usage and doses of herbal medicine in decoction and compounding, or variability in composition due to distinct product regions, subsequently risking the value of their diversity. As China, Japan, and Vietnam stress the importance of preserving the kinds and availability of herbal medicines in traditional medicine, they carefully established regulations that were compatible with the herbal medicines already in the marketplace, as reflected in the variation in upper limits shown in Fig. 4. Indeed, the preparation guidelines of JP18 state that the upper limits are set in consideration of natural content [24]. The same approach might have been taken in establishing the stipulations of CP2020 and VP5. However, the question of whether thresholds for ensuring safety should be determined based on the actual measurement of samples available in the market warrants further discussion. Setting a uniform upper limit that any herbal medicine should adhere to would be too loose from the perspective of safety, as Japan and Korea argued in the deliberations of ISO/TC249/WG1 [14].

(B) Stipulations for Individual Elements or Total Amount of Heavy Metals

IP2018 and JP18 required the total amount of heavy metals without determining individual elements only, although the other documents specified the individual determination of heavy metals. This difference is reflected in the test methods adopted in each document. Individual assay of each element is now becoming an international standard, as many countries and regions have revised or are revising pharmacopoeia following issuance of ICH-Q3D [8–10]. Individual assay is highly sensitive and scientifically valid [13]. However, IP2018 and JP18 adopted chemical methods for measurements of arsenic and total heavy metals. The analytical equipment for the individual determination of each element specified in the pharmacopoeia is unlikely affordable for smaller suppliers. If the guidance and monographs listed in a pharmacopoeia are mandatory, small suppliers may become unable to supply herbal medicines, in turn affecting the distribution of herbal medicines.

Given the difficulties in establishing “one-size-fits-all” regulations for all herbal medicines, further research and discussion on achieving the balance required between the diversity and standardization of herbal medicines are warranted. One feasible option is to set an upper limit with exceptions for some herbal medicines, which require specific consideration in upper limits values, as seen in the EP and KP. Furthermore, there are several economic obstacles to implementing standards for uniform assay methods for elemental impurities, as we mentioned above. To address this issue, we propose referencing the international standards for foods. Foods are not artificially synthesized, similarly to herbal medicines, and some countries classify herbal medicines into regulatory categories of health foods, functional foods and general food products [25]. Adherence to the Codex standard is recommended for international trade when taking SPS measures; however, adoption of the Codex standard into national regulations is not necessarily enforceable, as stated in the Agreement on the Application of SPS Measures [6]. Similarly, rigorous harmonization of herbal medicine regulations to the international standard should not be pursued, albeit that the standard should be used as the reference in the international marketplace. Rather, we should be prepared for national adoption of international standards when the obstacles to wide use of individual assays for elemental impurities are addressed, including the issues related to cost and efficiency. The Asia–Pacific Economic Cooperation-Life Sciences Innovation Forum-Regulatory Harmonization Steering Committee (APEC-LSIF-RHSC) has announced “regulatory convergence” as its vision for the future [26–28]. While harmonization refers to the state of uniformity of regulations in each country, convergence refers to the process of becoming more similar while acknowledging some differences, and not necessarily achieving uniformity [27]. Adapting a state of convergence would allow the setting of a permitted range of upper limits which takes account of regional differences in the compositions of herbal medicines originating from distinct cultivars, soils, climates, and seasonality. Thus, we suggest that a state of convergence to internationally agreed standards may be a potential goal for ensuring quality and diversity of herbal medicines. The International Herbal Pharmacopoeia currently compiled by WHO is expected to serve as a future international standard for convergence.

### Limitations

There is a limitation to this study. Several documents containing stipulations for herbal medicines other than major pharmacopoeias were not included in this study. Four other separate pharmacopoeias for traditional medicine, Ayurveda, Siddha, Unani, and Homeopathy, in India [29] and “The Japanese Standards for non-Pharmacopoeial Crude Drugs” for herbal medicines that are commercially available but not listed in JP [30] were excluded from analysis, in accordance with the predefined selection criteria.

## Conclusions

We studied pharmacopoeias, standards, and guidelines to summarize regulations on arsenic and heavy metals in herbal medicines, and to outline the status of harmonization. International standards such as the WHO guidelines and ISO standards have yet to be integrated and implemented in the regulations of all countries and regions we surveyed. The state of regulatory convergence to internationally agreed standards may be an achievable goal to balance international trade promotion and maintenance of both the diversity and safety of herbal medicines. The International Herbal Pharmacopoeia could serve as a potential integrated reference for future convergence.

## Supplementary Information

Below is the link to the electronic supplementary material.

Supplementary file1 (DOCX 13 KB)

## Data Availability

The authors confirm that the data supporting the findings of this study are available within the article and its supplementary materials.
