# Correction: Comparison of Regulations for Arsenic and Heavy Metals in Herbal Medicines Using Pharmacopoeias of Nine Counties/Regions

**DOI:** 10.1007/s43441-023-00539-9

**Published:** 2023-05-28

**Authors:** Isa Inada, Fumiyuki Kiuchi, Hisashi Urushihara

**Affiliations:** 1 Division of Drug Development and Regulatory Science, Faculty of Pharmacy, Keio University, 1-5-30 Shibakoen, Minato-Ku, Tokyo, 105-8512 Japan; 2 Division of Natural Medicines, Faculty of Pharmacy, Keio University, 1-5-30 Shibakoen, Minato-Ku, Tokyo, 105-8512 Japan

**Correctio to: Therapeutic Innovation & Regulatory Science** 10.1007/s43441-023-00532-2

In the Discussion section, the subheading "(B) Stipulations for Individual Elements or Total Amount of Heavy Metals" should be "(2) Stipulations for Individual Elements or Total Amount of Heavy Metals".

