# Supplementary material for: Comparison of Regulations for Arsenic and Heavy Metals in Herbal Medicines Using Pharmacopoeias of Nine Counties/Regions
Source: Ther Innov Regul Sci. 2023 May 18;57(5):963–74. doi: 10.1007/s43441-023-00532-2 (PMC10400675; doi:10.1007/s43441-023-00532-2)
Supplement: Supplementary file 1 — Supplementary file1 (DOCX 13 KB) [file 43441_2023_532_MOESM1_ESM.docx]

**Table S1** FHH participating countries and regions

| Participating country/region | Australia |
| --- | --- |
|  | China |
|  | Hong Kong |
|  | Japan |
|  | Korea |
|  | Singapore |
|  | Vietnam |
| Special member | Canada |

**Table S2** Countries and regions excluded from the target of study

| Reason for exclusion | Country/Region |
| --- | --- |
| There is no pharmacopoeia or corresponding documents. | Australia, Canada, Singapore |
| Pharmacopoeia has not been published in English. | Argentina, Austria, Belarus, Belgium, Bosnia and Herzegovina, Bulgaria, Chile, Croatia, Cyprus, Czech Republic, Denmark, Estonia, Finland, France, Germany, Greece, Hungary, Iceland, Indonesia, Iran, Ireland, Italy, Kazakhstan, Latvia, Lithuania, Luxembourg, Malta, Mexico, Montenegro, Netherlands, North Macedonia, Norway, Poland, Portugal, Romania, Russian Federation, Serbia, Slovakia, Slovenia, Spain, Sweden, Switzerland, Turkey, Ukraine, Eurasia |
| Pharmacopoeia does not list elemental impurities of herbal medicines. | Philippines, Thailand |
| Pharmacopoeia is difficult to obtain. | Africa, Egypt, Pakistan |
| Pharmacopoeia refers to the pharmacopoeia of another country or region for stipulations for elemental impurities in herbal medicines. | United Kingdom of Great Britain and Northern Ireland  (Stipulations on elemental impurities in herbal medicines are taken from European Pharmacopoeia) |
